# Speech and Language Errors during Awake Brain Surgery and Postoperative Language Outcome in Glioma Patients: A Systematic Review

**DOI:** 10.3390/cancers14215466

**Published:** 2022-11-07

**Authors:** Ellen Collée, Arnaud Vincent, Clemens Dirven, Djaina Satoer

**Affiliations:** Department of Neurosurgery, Erasmus MC-University Medical Center, Doctor Molewaterplein 40, 3015 GE Rotterdam, The Netherlands

**Keywords:** awake craniotomy, direct electrical stimulation, brain mapping, intraoperative language monitoring, speech and language errors, language outcome, glioma

## Abstract

**Simple Summary:**

Many glioma patients suffer from postoperative language problems after awake brain surgery, which have a negative effect on their quality of life. We investigated which language-related factors before and during surgery predicted language problems after surgery (language outcome). We found that language problems before surgery and word-finding and production problems during surgery were predictors for the language outcome. After surgery, the language problems that occurred most often were production deficits and spontaneous speech deficits. In conclusion, during surgery, word-finding problems and production errors should carry particular weight during decision making concerning the optimal onco-functional balance for a given patient, and spontaneous speech should be monitored. These new insights should be investigated further and may facilitate decision-making during surgery in the future, which can improve the procedure. This may improve the language outcome and ultimately the quality of life in this patient group.

**Abstract:**

Awake craniotomy with direct electrical stimulation (DES) is the standard treatment for patients with gliomas in eloquent areas. Even though language is monitored carefully during surgery, many patients suffer from postoperative aphasia, with negative effects on their quality of life. Some perioperative factors are reported to influence postoperative language outcome. However, the influence of different intraoperative speech and language errors on language outcome is not clear. Therefore, we investigate this relation. A systematic search was performed in which 81 studies were included, reporting speech and language errors during awake craniotomy with DES and postoperative language outcomes in adult glioma patients up until 6 July 2020. The frequencies of intraoperative errors and language status were calculated. Binary logistic regressions were performed. Preoperative language deficits were a significant predictor for postoperative acute (OR = 3.42, *p* < 0.001) and short-term (OR = 1.95, *p* = 0.007) language deficits. Intraoperative anomia (OR = 2.09, *p* = 0.015) and intraoperative production errors (e.g., dysarthria or stuttering; OR = 2.06, *p* = 0.016) were significant predictors for postoperative acute language deficits. Postoperatively, the language deficits that occurred most often were production deficits and spontaneous speech deficits. To conclude, during surgery, intraoperative anomia and production errors should carry particular weight during decision-making concerning the optimal onco-functional balance for a given patient, and spontaneous speech should be monitored. Further prognostic research could facilitate intraoperative decision-making, leading to fewer or less severe postoperative language deficits and improvement of quality of life.

## 1. Introduction

The standard treatment for patients with gliomas in eloquent brain areas is resection via awake craniotomy with direct electrical stimulation (DES) [1]. This procedure results in a larger extent of resection while maintaining postoperative neurological and cognitive function [1,2,3].

During stimulation of language areas (i.e., language mapping) and resection, various (temporary) speech errors and language errors (i.e., paraphasias, henceforth called errors) can be elicited. Some examples are anomia (word-finding difficulty), speech arrest, dysarthria (articulation difficulty) comprehension errors, semantic errors (related in meaning, such as “dog” for “cat”) and phonemic errors (substitution of sound(s), such as “lorse” for “horse”). These errors indicate that the corresponding language function is (at least partly) localized in that brain area [4,5] and that this area thus must be maintained or handled with caution during surgery.

Even though these errors and language errors in general are monitored carefully during surgery, the postoperative language outcome is often suboptimal. About 50% of the patients suffer from postoperative aphasia [6]. Different aphasic disturbances can occur, such as deficits in word-finding [7] and verbal fluency [8]. It is argued that these postoperative deficits are often transient [9]. However, some language problems, such as impairments in spontaneous speech [10] and verbal fluency [8], can persist until 1 year after surgery, which can have strong negative effects on the quality of life of the patient [11].

Various (preoperative) clinical variables can influence the language outcome of glioma patients after awake surgery, such as tumor characteristics. The risk of postoperative cognitive decline (including language) is reported to be increased by a larger tumor size [12] and, specifically for postoperative language decline only, by a tumor location in or near a language area [13,14]. Furthermore, postoperative language decline is often found to be associated with low-grade tumors [15], while postoperative cognitive improvement (including language) is found to be associated with high-grade tumors [12].

It was also found that the risk of postoperative language deficits is higher when preoperative language deficits are already present [16,17,18], when a suboptimal (but still within average range) score for object naming is found [17] and when seizures in combination with language deficits occur [18]. A marker of preoperative resting-state brain activity as measured by electroencephalography (EEG) (slow-wave activity in the theta band)has also been reported to predict postoperative language outcomes [19].

Additionally, intraoperative variables can also affect the language outcome. An association between the postoperative language outcome and the intraoperative scores of the Pyramid and Palm Tree Test (semantic test) was found [20]. Furthermore, relations between postoperative (transient) language deficits and the occurrence of intraoperative positive stimulation sites during language mapping were found within the tumor [17] and at the subcortical level using naming and comprehension tasks [16,21].

Moreover, a few studies observed a parallel between the occurrence of certain intraoperative language errors and postoperative aphasia syndrome, such as intraoperative problems in spontaneous speech and postoperative SMA syndrome or dynamic aphasia [9] as well as intraoperative phonemic errors and postoperative conduction aphasia [22]. However, the possible prognostic link between different intraoperative speech and language errors and the language outcome, is not clear.

Therefore, we aim to systematically review different intraoperative speech and language errors and the perioperative language status. Additionally, for the first time, we aim to investigate the potential prognostic relation between different intraoperative speech and language errors and (1) the occurrence of postoperative language deficits as well as (2) the type of postoperative language outcome defined by the linguistic modality (production, comprehension, reading and writing), aphasia syndrome (e.g., Broca’s aphasia, and conduction aphasia), linguistic level (phonology, semantics and morpho-syntax) or another level (e.g., articulation, spontaneous speech, speech apraxia and word-finding). These findings could be informative in terms of prognostics and providing patient information, and they may guide awake tumor resection in the future.

## 2. Materials and Methods

The details of the protocol for this systematic review were registered in the PROSPERO database (CRD42020196727) and divided into two: (1) intraoperative speech and language errors and brain locations and (2) this current article’s focus of intraoperative speech and language errors and language outcome.

### 2.1. Study Selection

A systematic search of five online databases (Embase, Medline Ovid, Web of Science, Cochrane and Google Scholar) was performed in line with the PRISMA statement guidelines [21] (for the search terms, see Text S1). Publication dates were included up until 6 July 2020. The search was performed by a reviewer (E.C.) in collaboration with a biomedical information specialist from the Erasmus Medical Centre Medical Library. Two senior co-authors were consulted for difficult cases (A.V. and D.S.).

### 2.2. Inclusion and Exclusion Criteria 

**Figure 1:**
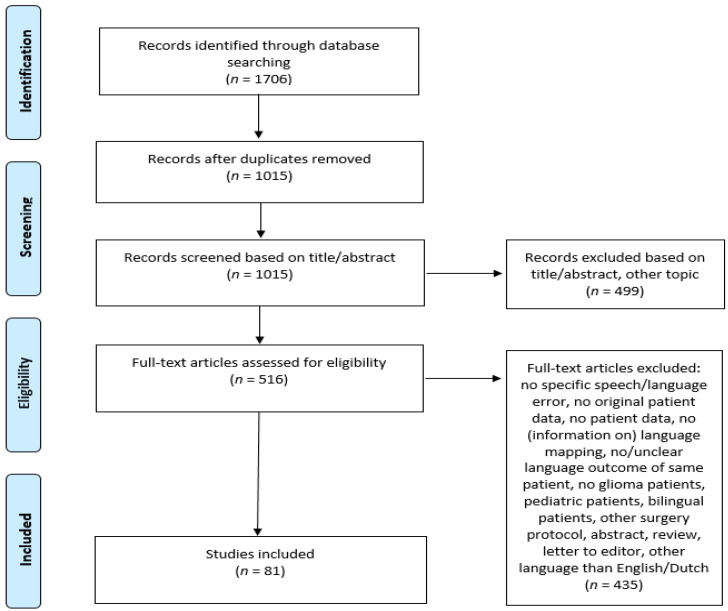
PRISMA flowchart of total records identified through searching of databases.

All articles reporting on speech and language errors (in detail) during awake craniotomies with DES (during DES or resection) in adult monolingual (≥18 years) patients with low- and high-grade gliomas (WHO grade II–IV), in combination with a postoperative language outcome, were included. Articles were excluded for multiple reasons (see Figure 1), such as the intraoperative language errors not being clearly specified. The PRISMA flowchart can be found in Figure 1.

### 2.3. Data Extraction and Organization

The number of patients, tumor grade, tumor location, speech and language errors and perioperative language status were extracted from the eligible studies. Language status was mainly based on clinical observations and, in some cases, based on standardized tests and reports by patients. Language status was categorized as the occurrence of language deficits: yes, no or unknown (unknown meaning that no information was reported on possible language deficits). In addition, the specific intraoperative speech and language errors were grouped into categories (see Table 1) based on linguistic modality (comprehension, production, reading and writing) or the linguistic level or other levels (articulation, morpho-syntax, phonology, semantics, spontaneous speech, speech apraxia and word finding). The error categories that occurred <10 times per outcome option (postoperative language deficits (yes, no or unknown)) were grouped under “other errors” (see Table S1 for more information about these errors).

Considering that much variation occurred between articles, the time points were grouped as follows: (T0) preoperatively and postoperatively: (T1) acute (1–10 days), (T2) sub-acute (≥2 weeks–3 months), (T3) short-term follow-up (≥3–8 months) and (T4) long-term follow-up (12–15 months).

The language outcome (i.e., postoperative language status) reported at these time points was first defined by the presence of language deficits (yes, no or unknown). Secondly, the type of outcome was determined when possible. General descriptions of the outcomes (e.g., language problems or aphasia) were not categorized. All other outcomes were grouped according to the modality or aphasia syndrome (see Table 2). As a next step, (part of) the outcomes were also grouped according to linguistic level or other levels if possible (see Table 2). Note that the outcomes often included multiple deficits, of which all individual complaints could not always be categorized.

### 2.4. Analyses

First, the frequencies of all individual intraoperative errors and language statuses (language deficits: yes, no or unknown) were calculated. Second, the distribution of the different intraoperative language error categories (six types (see Table 1)) and language outcomes per postoperative time point were inspected. Only the time points that included intraoperative error categories that occurred >10 times per outcome option were selected for statistical analyses, which were T1 (acute) and T3 (short-term follow-up). Binary logistic regressions with postoperative language deficits (yes or no) as the dependent variable and preoperative language deficits (yes, no or unknown) and intraoperative speech and language error categories (six types) as categorical predictors was performed for T1 and T3. The reference categories for the categorical predictors were no preoperative language problems and intraoperative speech arrest. Speech arrest was chosen due to the high frequency in the data. Based on the regression models, the marginal probabilities were calculated.

Third, the distribution of the different intraoperative language error categories and language outcome types (linguistic modality or aphasia syndrome and linguistic level or other level) per postoperative time point were inspected. Only T1 included enough data points in total (≥100) to conduct statistical analyses. Instances of <5 for some combinations occurred (intraoperative errors × linguistic modality or aphasia syndrome or linguistic level or other factor). Therefore, chi-squared tests with Monte Carlo simulation were performed to examine the relation between intraoperative error categories and postoperative linguistic modality or aphasia syndrome and linguistic level or other levelat T1. Intraoperative error category 6 (“other errors”) was excluded here, since we were interested in specific errors for this analysis. No statistical post hoc analysis could be performed due to a lack of sufficient data points per outcome type. Therefore, the crosstabs were used to describe these data.

## 3. Results

In all, 1706 articles were found. After duplications were removed, 1015 remained. Of these articles, 499 were excluded based on title and abstract, and 516 articles were reviewed in full text, of which 435 were excluded (see Figure 1 for reasons), while 81 were included (see references [9,23,24,25,26,27,28,29,30,31,32,33,34,35,36,37,38,39,40,41,42,43,44,45,46,47,48,49,50,51,52,53,54,55,56,57,58,59,60,61,62,63,64,65,66,67,68,69,70,71,72,73,74,75,76,77,78,79,80,81,82,83,84,85,86,87,88,89,90,91,92,93,94,95,96,97,98,99,100,101,102] for all included articles). The collected information from the articles is shown in Table 3. The tumor grade and location were based on the total number of errors and not the total number of patients.

### 3.1. Intraoperative Speech and Language Errors and Language Status

Fourteen different intraoperative errors were reported (see Table 4), of which some occurred frequently (anomia or speech arrest >20%) and some occurred infrequently (irrelevant paraphasia, neologisms or speech apraxia <0.5%).

**Figure 2:**
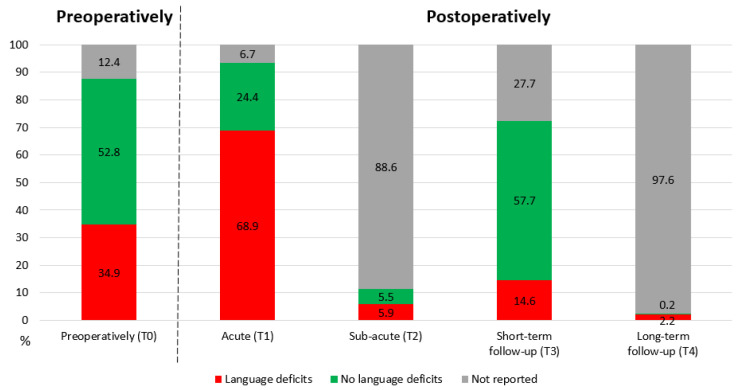
Language status in the preoperative (T0) and postoperative phases (T1–T4).

Language status was reported in more than 70% of the instances at T0, T1 and T3 but only in less than 12% at T2 and T4 (see Figure 2). Preoperatively, language deficits were reported in 34.9% of the cases. This increased to 68.9% at T1 and then decreased to 14.6% at T3.

### 3.2. Relation to Postoperative Acute and Short-Term Language Outcome (T1 and T3)

Preoperative language deficits (OR = 3.42, 95% CI 2.101–5.580, *p* < 0.001), intraoperative anomia (OR = 2.09, 95% CI 1.154–3.791, *p* = 0.015) and intraoperative production errors (OR = 2.06, 95% CI 1.141–3.716, *p* = 0.016) were significant predictors for postoperative language deficits at T1 (see Table 5).

The marginal probabilities of the occurrence of postoperative language deficits at T1 when intraoperative anomia occurred were 75.5% and 91.3%, and when intraoperative production errors occurred, they were 75.2% and 91.2% (without and with preoperative language deficits, respectively).

**Figure 3:**
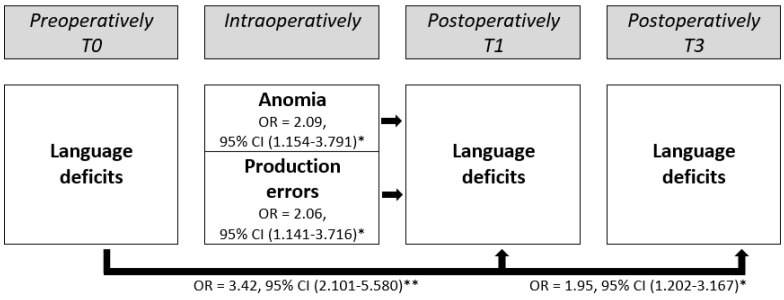
Significant preoperative and intraoperative predictors for postoperative language deficits at T1 (*n* = 589) and T3 (*n* = 456). T0 = preoperatively and postoperatively, T1 = 1–10 days, T3 = ≥3–8 months, OR = odds ratio and CI = confidence interval. This figure describes the binary logistic regression (T1 and T3) with the predictors of preoperative language status, intraoperative speech and language error categories, where the dependent variable is postoperative language outcome and reference categories are no preoperative language deficits and intraoperative speech arrest. * = *p* < 0.05. ** = *p* < 0.001.

Additionally, the preoperative language deficits (OR = 1.95, 95% CI (1.202–3.167), *p* = 0.007) and “unknown” preoperative language deficits (i.e., when no information was reported on possible preoperative language deficits, OR = 0.18, 95% CI (0.042–0.781) and *p* = 0.022) were significant predictors for postoperative language deficits at T3 (see Table 5). The results of these regressions are summarized in Figure 3 (excluding the “unknown” preoperative language deficits predictor for T3 for simplicity).

### 3.3. Relation to the Type of Postoperative Acute Language Outcome (T1)

Chi-square tests with Monte Carlo simulations showed that the relation between the intraoperative error categories and postoperative language outcome at T1 in terms of linguistic modality or aphasia syndrome (*p* < 0.001) and linguistic level or other level (*p* < 0.001) was significant.

The most frequently observed postoperative language deficits in terms of linguistic modality or aphasia syndrome were production deficits (*n* = 205, Table 6). Postoperative production deficits were most often observed after the occurrence of all intraoperative error categories, compared toother postoperative deficits in terms of linguistic modality or aphasia syndrome. Postoperative production deficits were observed, ranked by frequency, after the occurrence of intraoperative production errors (*n* = 73), anomia (*n* = 46), speech arrest (*n* = 41), semantic errors (*n* = 23) and phonemic errors (*n* = 22).

The most frequently observed postoperative language deficits, in terms of linguistic level or otherlevel, were deficits in spontaneous speech (*n* = 41) and articulation (*n* = 38, Table 6). Postoperative spontaneous speech deficits were observed after the occurrence of all intraoperative error categories: intraoperative anomia (*n* = 12), production errors (*n* = 10), speech arrest (*n* = 9), phonemic errors (*n* = 8) and semantic errors (*n* = 2). Within the categories of intraoperative anomia, speech arrest and phonemic errors, postoperative spontaneous speech deficits were observed most frequently out of all postoperative deficits in terms of linguistic level or otherlevel. Postoperative articulation deficits were most often observed after intraoperative production deficits (*n* = 18). Postoperative semantic deficits were most frequently observed after intraoperative semantic errors (*n* = 7).

## 4. Discussion

For the first time, we performed a systematic search of the literature to investigate the occurrence of different intraoperative speech and language errors and the perioperative language status, as well as their relation.

### 4.1. Intraoperative Speech and Language Errors and Language Status

Fourteen different error types were reported, of which some occurred frequently (e.g., speech arrest and anomia) and some occurred infrequently (e.g., irrelevant paraphasia, neologisms and speech apraxia). Language status was often reported preoperatively and postoperatively in the acute and short-term follow-up phases (T1 and T3, respectively) but not in the sub-acute or long-term follow-up phases (T2 and T4, respectively). Most language deficits occurred postoperatively in the acute phase (T1) and were resolved by the short-term follow-up (T3). This pattern of postoperative transient language deficits is well-known [9,103].

However, postoperative language deficits still occurred in 14.6% of cases at the short-term follow-up (T3). Unfortunately, it is unknown whether these deficits were still present at the long-term follow-up (T4), since only 2.2% of cases reported an outcome at this time point. Considering that language deficits can still be present a year after surgery [10], a longer follow-up period is necessary for this patient group, as also indicated by Satoer et al. [104].

### 4.2. Relation to Postoperative Acute and Short-Term Language Outcome (T1 and T3)

The results from the regressions and the marginal probabilities confirmed that the chance of postoperative language deficits in the acute and short-term follow-up phases (T1 and T3, respectively) was higher when preoperative language deficits were present. This is in line with previous studies [16,17,18]. Language networks may be less sensitive to postoperative neuroplasticity, since this reorganization ability may already have been exhausted preoperatively [17], caused by slow tumor growth.

Surprisingly, we also found that “unknown” preoperative language deficits (i.e., when no information was reported on possible preoperative language deficits) was a significant predictor for postoperative short-term language deficits (T3). This may be due to the size and nature of this data group. A fairly large part (11.8%) of all preoperative deficits in this analysis (*n* = 237) was marked as these “unknown” deficits. This part presumably consisted of patients with and without language deficits, resulting in mixed results. This underlines the importance of obtaining information on possible preoperative language deficits, considering that the predictions for the outcome were more distinct when this information was available. 

Furthermore, the findings suggest that the occurrence of intraoperative anomia and production errors were also predictors for postoperative language deficits at the acute phase (T1), probably mapping onto multiple broader semantic and phonological networks [105,106,107,108]. These results underline the importance of object naming and production tests (e.g., repetition and verbal diadochokinesis) during surgery (see Section 4.4).

### 4.3. Relation to the Type of Postoperative Acute Language Outcome (T1)

Intraoperative error categories and postoperative outcome in the acute phase (T1) in terms of linguistic modality or aphasia syndrome (comprehension, production, reading, Broca’s aphasia and conduction aphasia) and linguistic level or other level (phonology, semantics, morpho-syntax, articulation, spontaneous speech, speech apraxia and word-finding) were related. Our descriptive results show that the most frequently observed postoperative deficits were production deficits (in terms of modality or aphasia syndrome) and spontaneous speech deficits (in terms of the linguistic or other levels). Both of these postoperative deficits were observed after the occurrence of all intraoperative error categories. This shows that language production was most often impaired at the acute phase (T1). Additionally, it confirms that multiple linguistic levels are necessary for intact spontaneous speech production and that the disturbance of at least one component results in spontaneous speech output deficits. Considering that the articles often did not provide detailed information, and multiple types of spontaneous speech deficits were grouped, we could not determine whether the spontaneous speech deficits in this study stemmed from problems at the word level or sentence level (i.e., grammatical difficulties). Moreover, it is not clear whether the spontaneous speech deficits in this study could be defined as dynamic aphasia. Dynamic aphasia is a disorder characterized by reduced spontaneous speech and speech initiation while naming, repetition and comprehension are intact [109]. This disorder is generally associated with frontal lesions in the supplementary motor area (SMA) [23,110]. However, perhaps damage to other areas beyond the SMA can result in these difficulties as well, considering that spontaneous speech deficits occurred the most often postoperatively out of all errors (in terms of linguistic or other levels), even though many tumor locations in this study were not in the SMA (tumor locations: 41% in the frontal lobe, of which a smaller unknown part would be the SMA specifically, 28% in other lobes and 31% in combined lobes).

Even though variation occurred, the type of intraoperative error and type of postoperative language deficit defined by the linguistic modality or linguistic level were sometimes similar. For example, intraoperative production errors (e.g., dysarthria or slurred speech) were most frequently followed by postoperative production deficits (linguistic modality). Additionally, intraoperative semantic errors (i.e., errors related in meaning, such as “cat” for “dog”) were most frequently followed by postoperative semantic deficits (linguistic level). This can be explained by the fact that resection is performed close to a language area responsible for (a) specific linguistic function(s), such as semantics. Working close to this area can then result in disruption of the semantic system, logically resulting in both intraoperative and postoperative semantic errors.

### 4.4. Clinical Relevance

Our results suggest that, apart from the obvious speech arrest, production errors and anomia are important errors during surgery, since the occurrence of these error categories appeared to be linked to postoperative language deficits. Therefore, these errors should be monitored carefully. Anomia can be elicited with a task such as object naming, which is one of the most widely used tasks during awake surgery. Production errors can also be elicited with an object naming task but also with more specific articulation tasks such as word repetition and verbal diadochokinesis. The occurrence of these errors during surgery should carry particular weight during decision-making concerning the optimal onco-functional balance for each individual patient. The results concerning the relation between the type of intraoperative error and the type of postoperative language deficit can be used for preparing and informing the patient.

Out of all postoperative language deficits (in terms of linguistic or other levels), postoperative spontaneous speech deficits were observed most often. These deficits occurred after all intraoperative speech and language errors, thus arising from disruptions at multiple linguistic levels. Spontaneous speech is a central part of everyday communication and quality of life, and it is thus crucial to preserve it. Therefore, it should be tested during surgery. Unfortunately, spontaneous speech is not often reported to be monitored during awake brain surgery [109]. Spontaneous speech can be elicited in an interview setting with preoperatively defined topics, such as work or hobbies, as described by Satoer et al. [111]. When the spontaneous speech deteriorates in terms of initiation of conversation, fluency of speech or via the occurrence of speech and language errors, tasks targeting a specific linguistic level can be used to further investigate the level of deterioration from, for example, the Dutch Linguistic Intraoperative Protocol (DuLIP) [24]. For example, when a phonemic paraphasia occurs in spontaneous speech, a word repetition task can be selected [109]. In this way, spontaneous speech can be used to guide language monitoring and resection.

Isolated language tasks can also be used to elicit spontaneous speech in context [109], such as sentence completion from DuLIP. In this task, patients have to complete a sentence with either one or two words (closed context) or with a constituent (broad context). The broad context task is especially useful for monitoring spontaneous speech in context, since it requires forming a grammatically and semantically correct sentence (“At 5 o’clock […] the neighbor drives to work”). Sentence completion can be used during stimulation and resection. Another task for eliciting spontaneous speech in context during surgery is the sentence generation task [112], in which pictures of geometrical shapes are shown and the spatial relation has to be described (“The blue triangle is above the red circle”). We advise monitoring spontaneous speech (in context) structurally during surgery and using it as guidance during awake craniotomy.

### 4.5. Limitations and Future Research

A limitation of this study is that the articles varied greatly in how detailed they were when reporting on intraoperative speech and language errors (e.g., nature of error, definitions and type of errors), language status and at which time points they were reported. Information was often missing or unclear and could therefore not be included, which also meant that no statistical analysis was possible for certain time points. Language status was often based on clinical observations instead of standardized tests, resulting in a subjective outcome that may not be fully accurate. Another limitation is that each intraoperative error was coded separately and not per patient, resulting in a simplification of clinical practice, considering that multiple different errors can be elicited in one patient.

Even though more intraoperative tasks are available, such as DuLIP [24], many articles only used object naming during surgery. Object naming often elicits anomia, while other errors (e.g., syntactic errors) are less or not likely to be elicited during this task. Therefore, anomia may have been overrepresented, while other errors may have been missed. Further research should focus on the sensitivity of different standardized language tasks and their relation to intraoperative speech and language errors.

Due to many missing data, no statistical post hoc analysis could be performed to investigate the relation between the type of intraoperative speech and language error and the type of postoperative language outcome. Future research should explore this further. This could help with informing the patient better and selecting more specific therapies after surgery based on a specific linguistic modality or level.

This study emphasizes the importance of spontaneous speech monitoring during surgery. Considering that intraoperative spontaneous speech has not been investigated in depth before, future research should zoom in on the properties of it, including the different speech and language errors it contains and in which way it changes over time in the perioperative period. 

Lastly, considering that this study was based on many different articles which all reported differently, we underline the importance of intraoperative anomia and production errors and their relation to postoperative language deficits, but we cannot provide a critical cut-off point for when a functional boundary is truly reached and resection should be stopped based on the current data (e.g., after the occurrence of x times anomia). However, more in-depth research could possibly determine these critical cut-off points by constructing a prognostic severity scale for intraoperative speech and language errors on postoperative language outcome. This could be used to define functional boundaries even more accurately during awake tumor resection, which could result in less postoperative language deficits, possibly leading to improvement in a patient’s quality of life.

## 5. Conclusions

This systematic review investigated the relation between speech and language errors during awake craniotomy and the postoperative language outcomes of glioma patients. Our results suggest that the occurrence of preoperative language deficits, intraoperative anomia and intraoperative production errors are predictors for postoperative language deficits. These intraoperative errors should carry particular weight during decision making concerning the optimal onco-functional balance for a given patient during surgery. Spontaneous speech should also be monitored carefully during surgery, and it can be used as guidance during resection. Investigating the prognostic value of intraoperative speech and language errors on postoperative language outcomes further may improve language monitoring, which could potentially result in a reduction in postoperative language deficits and the improvement of quality of life in patients undergoing awake craniotomy.

## Figures and Tables

**Table 1:** Intraoperative speech and language errors taken from the articles and their corresponding categories (in bold).

No	Speech and Language Errors	No	Speech and Language Errors
**1**	**Anomia or word-finding difficulties**	**4**	**Semantic errors**
	Anomia		Non-speech semantic processing problem
	Circumlocutions		Semantic association disturbance or error
	Naming delay or delayed word retrieval		Semantic comprehension error
	Word-finding, searching or retrieval difficulties		Semantic disorder, deficit or aphasia
**2**	**Phonemic errors**		**Semantic disturbance or error**
	Phonemic disturbance		Semantic jargon aphasic language
	Phonemic paraphasias in writing		Semantic paraphasias in writing
	Phonemic speech error or paraphasia		Semantic speech error or paraphasia
	Phonological paraphasia	**5**	**Speech arrest**
	Phonological processing or disturbance	**6**	**Other errors**
**3**	**Production errors**		Comprehension errors *
	Anarthria		Irrelevant paraphasia
	Articulatory difficulty		Morpho-syntactic errors *
	Dysarthria		Neologism
	Hesitation		Perseveration
	Slow speech		Reading errors *
	Slurred speech		Speech initiation difficulties *
	Speech delay		Speech apraxia
	Stammering		Writing errors *
	Stuttering		

Categories are printed in bold, No = number. Note: a paraphasia is a language error. * See List S1 for more information about the types of errors grouped into this category.

**Table 2:** The language outcome categories defined by (1) linguistic modality or aphasia syndrome and (2) linguistic level or other factors.

Language Outcome Categories	Language Outcome Categories
**1.A. Linguistic modality**	**1.B. Aphasia syndrome**
Comprehension	Broca, motor, or expressive aphasia
Production	Conduction aphasia
Reading	-
**2.A. Linguistic level**	**2.B. Other**
Phonology	Articulation
Semantics	Spontaneous speech *
Morpho-syntax	Speech apraxia
-	Word finding

* Including speech fluency deficits, SMA syndrome deficits or mutism. Categories are printed in bold.

**Table 3:** Overview of information from included articles.

Information from Incluced Articles	All Articles
Total articles	81
Total errors	631
Number of awake patients in articles (range)	1–107
Tumor Grade	
Low grade	452
High grade	118
Not stated	61
Tumor Location: Hemisphere	
Left	552
Right	75
Not stated	4
Tumor Location: Lobe	
Frontal	256
Parietal	57
Temporal	87
Occipital	3
Insular	32
Combination	196

**Table 4:** The occurrence of different intraoperative speech and language errors in absolute numbers and percentages, ranked by frequency (*n* = 631).

Individual Error	Error Category	Absolute Number	Percentage
Anomia	Anomia	132	20.9%
Speech arrest	Speech arrest	132	20.9%
Production errors	Production errors	124	19.7%
Semantic errors	Semantic errors	88	13.9%
Phonemic errors	Phonemic errors	76	12.0%
Perseveration	Other errors	23	3.6%
Reading errors	Other errors	20	3.2%
Morpho-syntactic errors	Other errors	15	1.1%
Writing errors	Other errors	7	0.8%
Speech initiation difficulties	Other errors	5	0.8%
Comprehension errors	Other errors	5	0.8%
Irrelevant paraphasia	Other errors	2	0.3%
Neologism	Other errors	1	0.2%
Speech apraxia	Other errors	1	0.2%

**Table 5:** Binary logistic regression models of predictors for language outcome at T1 (*n* = 589) and T3 (T3, *n* = 456).

(Possible) Predictors	T1			T3		
	B (95% CI)	S.E.	Exp (B)	B (95% CI)	S.E.	Exp (B)
Pre deficits (yes)	1.231 ** (2.101–5.580)	0.249	3.424	0.669 * (1.202–3.167)	0.247	1.951
Pre deficits (unknown)	−0.530 (0.346–1.002)	0.271	0.589	−1.704 * (0.042–0.781)	0.744	0.182
Intra anomia	0.738 * (1.154–3.791)	0.303	2.092	0.614 (0.873–3.914)	0.383	1.848
Intra phonemic errors	0.381 (0.754–2.840)	0.338	1.463	0.540 (0.741–3.973)	0.428	1.716
Intra production errors	0.722 * (1.141–3.716)	0.301	2.059	-0.192 (0.363–1.875)	0.419	0.825
Intra semantic errors	0.332 (0.731–2.657)	0.329	1.394	−0.098 (0.376–2.190)	0.450	0.907
Intra other errors	0.064 (0.542–2.097)	0.345	1.066	0.545 (0.723–4.114)	0.444	1.725
Constant	0.385	0.213	1.470	−1.783	0.327	0.168

T1 = acute: 1–10 days postoperatively, T3 = short-term follow-up: ≥3–8 months postoperatively, pre = preoperative, intra = intraoperative, unknown indicates that no information was reported about possible preoperative deficits, and reference categories are no preoperative language deficits and intraoperative speech arrest. * = *p* < 0.05. ** = *p* < 0.001. Significant values are printed in bold.

**Table 6:** The absolute frequencies of postoperative language deficits defined by linguistic modality or aphasia syndrome and linguistic level or other levelafter different intraoperative speech and language error categories.

Postoperative Language Deficits	Intraoperative Speech and Language Errors					
	Anomia	Phonemic Errors	Production Errors	Semantic Errors	Speech Arrest	Total
By linguistic modality or aphasia syndrome						
Production	46	22	73	23	41	205
Comprehension	3	3	0	3	4	13
Reading	16	8	1	7	8	40
Conduction aphasia	3	5	2	0	2	12
Broca’s aphasia	3	0	9	0	10	22
Total	71	38	85	33	65	292
By linguistic level or other level						
Articulation	6	3	18	2	9	38
Morpho-syntax	3	3	0	6	5	17
Phonology	0	5	3	1	0	9
Semantics	2	1	5	7	0	15
Spontaneous speech	12	8	10	2	9	41
Speech apraxia	0	0	2	2	0	4
Word finding	1	1	2	1	4	9
Total	24	21	40	21	27	133

## Data Availability

The data presented in this study are available on request from the corresponding author.

## Supplementary Materials

The following supporting information can be downloaded at https://www.mdpi.com/article/10.3390/cancers14215466/s1. Text S1: Search terms. Table S1: Intraoperative speech and language errors and their categories.
