# Supplementary material for: Speech and Language Errors during Awake Brain Surgery and Postoperative Language Outcome in Glioma Patients: A Systematic Review
Source: Cancers (Basel). 2022 Nov 7;14(21):5466. doi: 10.3390/cancers14215466 (PMC9658495; doi:10.3390/cancers14215466)
Supplement: Supplementary file 1 [file cancers-14-05466-s001.zip › cancers-1982645-supplementary.pdf]

Supplementary Materials: Speech and Language Errors During Awake Brain Surgery and Postoperative Language Outcome in Glioma Patients: A Systematic Review

Ellen Collée, Arnaud Vincent, Clemens Dirven and Djaina Satoer

Text S1: Search terms

1.1. Embase.com

('surgery'/de OR surgery:lnk OR 'surgical technique'/de OR 'neurosurgery'/exp OR 'cancer surgery'/de OR 'intraoperative period'/de OR 'brain mapping'/de OR 'electrostimulation'/de OR 'neuromonitoring'/de OR 'glioma'/exp OR 'brain tumor'/exp OR (map* OR language*-monitor* OR neuromonitor* OR neuro-monitor* OR craniotom* OR surg* OR neurosurg* OR operat* OR intraoperat* OR anesthes* OR resect* OR electrostimulation* OR electro-stimulation* OR glioma* OR glioblastoma* OR astrocytoma* OR oligodendroglioma* OR ((intracranial* OR brain*) NEAR/3 (neoplas* OR tumor* OR tumour* OR cancer*))):ab,ti,kw) AND ('wakefulness'/de OR (awake* OR waking* OR wake*):ab,ti,kw) AND ('speech disorder'/exp OR 'perseveration'/de OR 'language ability'/de OR 'language disability'/de OR 'linguistics'/exp OR 'language processing'/de OR (aphasi* OR paraphasi* OR dysphasi* OR perseveration* OR anomia* OR apraxia* OR dysarthria* OR semantic* OR neologism* OR linguistic* OR ((language* OR speech*) NEAR/3 (error* OR arrest* OR perform* OR mistake* OR disturbance* OR abilit* OR disabilit* OR dysfunction* OR disorder* OR process*))):ab,ti,kw)

1.2. Medline Ovid

(surgery.fs. OR Neurosurgery/ OR exp Neurosurgical Procedures/ OR Intraoperative Period/ OR Brain Mapping/ OR exp Glioma/ OR exp Brain Neoplasms/ OR (map* OR language*-monitor* OR neuromonitor* OR neuro-monitor* OR craniotom* OR surg* OR neurosurg* OR operat* OR intraoperat* OR anesthes* OR resect* OR electrostimulation* OR electro-stimulation* OR glioma* OR glioblastoma* OR astrocytoma* OR oligodendroglioma* OR ((intracranial* OR brain*) ADJ3 (neoplas* OR tumor* OR tumour* OR cancer*))).ab,ti,kf.) AND (Wakefulness/ OR (awake* OR waking* OR wake*).ab,ti,kf.) AND (exp Speech Disorders/ OR Language Disorders/ OR Anomia/ OR Linguistics/ OR (aphasi* OR paraphasi* OR dysphasi* OR perseveration* OR anomia* OR apraxia* OR dysarthria* OR semantic* OR neologism* OR linguistic* OR ((language* OR speech*) ADJ3 (error* OR arrest* OR perform* OR mistake* OR disturbance* OR abilit* OR disabilit* OR dysfunction* OR disorder* OR process*))).ab,ti,kf.)

1.3. Web of Science

TS=(((map* OR language*-monitor* OR neuromonitor* OR neuro-monitor* OR craniotom* OR surg* OR neurosurg* OR operat* OR intraoperat* OR anesthes* OR resect* OR electrostimulation* OR electro-stimulation* OR glioma* OR glioblastoma* OR astrocytoma* OR oligodendroglioma* OR ((intracranial* OR brain*) NEAR/2 (neoplas* OR tumor* OR tumour* OR cancer*)))) AND ((awake* OR waking* OR wake*)) AND ((aphasi* OR paraphasi* OR dysphasi* OR perseveration* OR anomia* OR apraxia* OR dysarthria* OR semantic* OR neologism* OR linguistic* OR ((language* OR speech*) NEAR/2 (error* OR arrest* OR perform* OR mistake* OR disturbance* OR abilit* OR disabilit* OR dysfunction* OR disorder* OR process*)))))

1.4. Cochrane Central Register of Controlled Trials

((map* OR (language* NEXT/1 monitor*) OR neuromonitor* OR neuro-monitor* OR craniotom* OR surg* OR neurosurg* OR operat* OR intraoperat* OR anesthes* OR resect*

OR electrostimulation* OR electro-stimulation* OR glioma* OR glioblastoma* OR astrocytoma* OR oligodendroglioma* OR ((intracranial* OR brain*) NEAR/3 (neoplas* OR tumor* OR tumour* OR cancer*))):ab,ti,kw) AND ((awake* OR waking* OR wake*):ab,ti,kw) AND ((aphasi* OR paraphasi* OR dysphasi* OR perseveration* OR anomia* OR apraxia* OR dysarthria* OR semantic* OR neologism* OR linguistic* OR ((language* OR speech*)

NEAR/3 (error* OR arrest* OR perform* OR mistake* OR disturbance* OR abilit* OR disabilit* OR dysfunction* OR disorder* OR process*))):ab,ti,kw)

1.5. Google Scholar

craniotomy|mapping|surgery|neurosurgery|intraoperative|resection|resected|electrostimulation|glioma|glioblastoma|astrocytoma|oligodendroglioma awake|waking|wakefulness

aphasia|paraphasia|dysphasia|"language|speech errors|performance|disorders|processing"

1.6. Medline (Ovid)

Neurosurgery/
Exp Brain Mapping/
Exp Glioma/
Wakefulness/
Exp Speech Disorders/

Table S1. Intraoperative speech and language errors and their categories.

No	Speech and Language Errors	No	Speech and Language Errors
**1**	**Anomia/word-finding difficulties**	**6**	**Other errors**
	Anomia		**Comprehension errors**
	Circumlocutions		Comprehension difficulty
	Naming delay/delayed word retrieval		Impaired comprehension
	Word-finding/searching/retrieval difficulties		Auditory comprehension difficulty
**2**	**Phonemic errors**		Word deafness
	Phonemic disturbance		**Reading errors**
	Phonemic paraphasias in writing		Alexia
	Phonemic speech error/paraphasia		Reading deficit/disturbances/impairment
	Phonological paraphasia		Reading arrest
	Phonological processing/disturbance		Delayed (comprehensive) reading
**3**	**Production errors**		**Speech initiation difficulties**
	Anarthria		Delayed speech initiation
	Articulatory difficulty		Initiation difficulty
	Dysarthria		SMA aphasia
	Hesitation		Difficulty with sentence completion
	Slow speech		Reduction of spontaneous speech
	Slurred speech		**(Morpho-)syntactic errors**
	Speech delay		Syntactic disorders
	Stammering		Syntactic gender error
	Stuttering		Morphological overregularization (verbs)
**4**	**Semantic errors**		Inflection errors
	Non-verbal semantic processing problem		**Perseveration**
	Semantic association disturbance/error		**Writing errors**
	Semantic comprehension error		Writing arrest
	Semantic disorder/deficit/aphasia		Alterations of letter shapes in writing
	Semantic disturbance/error		Writing drift
	Semantic jargon aphasic language		Spelling errors
	Semantic paraphasias in writing		**Verbal apraxia**
	Semantic speech error/paraphasia		**Irrelevant paraphasia**
**5**	**Speech arrest**		**Neologism**

No = number; All errors grouped together in category 6 "Other errors" are displayed separately. The categories are in bold.
